# Epigenetic Regulation of Human Vascular Calcification

**DOI:** 10.3390/genes16050506

**Published:** 2025-04-28

**Authors:** Lova Prasadareddy Kajuluri, Yugene Young Guo, Sujin Lee, Michael Christof, Rajeev Malhotra

**Affiliations:** 1 Cardiovascular Research Center, Heart and Vascular Institute, Mass General Brigham, Boston, MA 02114, USA; lkajuluri@mgh.harvard.edu (L.P.K.); yyguo@mgh.harvard.edu (Y.Y.G.); slee@mgh.harvard.edu (S.L.); 2 School of Arts and Sciences, University of Rochester, Rochester, NY 14627, USA; mchris25@u.rochester.edu

**Keywords:** vascular calcification, epigenetics, CVDs, VSMCs

## Abstract

Vascular diseases present a significant threat to human health worldwide. Atherosclerosis is the most prevalent vascular disease, accounting for the majority of morbidity and mortality globally. Vascular calcification is a dynamic pathological process underlying the development of atherosclerotic plaques and involves the phenotypic transformation of vascular smooth muscle cells (VSMCs) into osteogenic cells. Specifically, the phenotypic switch in VSMCs often involves modifications in gene expression due to epigenetic changes, including DNA methylation, histone modification, and non-coding RNAs. Understanding the role of these epigenetic changes in regulating the pathophysiology of vascular calcification, along with the proteins and pathways that mediate these changes, will aid in identifying new therapeutic candidates to enhance vascular health. This review discusses a comprehensive range of epigenetic modifications and their implications for vascular health and the development of vascular calcification.

## 1. Introduction

Cardiovascular diseases (CVDs) are the leading cause of morbidity and mortality worldwide, with an estimated 18 million deaths annually, 85% of which are due to myocardial infarction and stroke [1]. Accompanied by the increasing incidence of hypertension and type 2 diabetes in an aging population, the obesity epidemic has hindered our ability to address the worldwide burden of CVD despite advances in biomedical research [2]. Furthermore, the costs of CVD are projected to climb to approximately USD 1.1 trillion in the United States by 2050 [3]. Understanding the complex pathophysiology of CVD is essential to foster the development of novel therapies and reduce the burden of CVD.

Atherosclerosis is a disease characterized by the progressive buildup of plaque in the walls of arteries, ultimately leading to tissue ischemia [4,5]. Coronary artery disease (CAD), carotid artery disease, and peripheral artery disease are three common forms of atherosclerosis that impact blood supply to the heart, brain, and lower extremities, respectively. Vascular calcification occurs in atherosclerotic plaques due to deposition of calcium phosphate minerals in the extracellular matrix [6]. The crucial step leading to vascular calcification is the phenotypic alteration of VSMCs in the medial layer of blood vessels into osteogenic cells. VSMCs typically exist in a contractile, non-proliferative phenotype. However, in response to various stimuli, they undergo a phenotypic switch with increased proliferation and reduced contractility. Additionally, vascular aging, along with risk factors such as diabetes, hypertension, metabolic disorders, and inflammation, further contributes to this pathological transformation of VSMCs [7,8].

**Figure 1:**
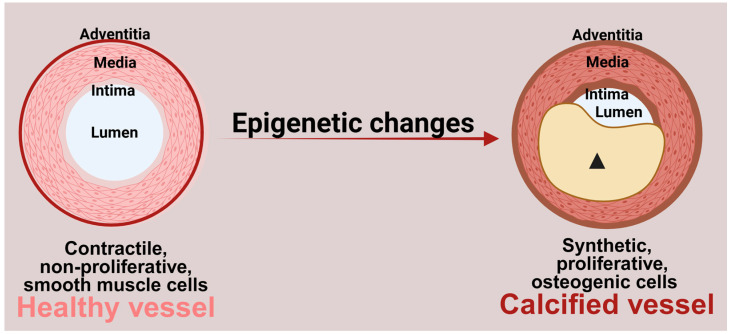
Role of epigenetic changes in vascular calcification. Epigenetic changes contribute to the transition of contractile, non-proliferative smooth muscle cells to highly proliferative, osteogenic cells. These osteogenic cells secrete calcium phosphate minerals into the extracellular matrix, resulting in vascular calcification. (Arrowhead: calcified plaque). Figure made in Bio-Render.

The molecular mechanisms involved in this phenotypic transition include changes in gene expression that lead to the upregulation of bone-related proteins, such as runt-related transcription factor 2 (RUNX2), bone morphogenetic proteins (BMPs), alkaline phosphatase (ALP), osteocalcin, osteopontin, etc., and downregulation of smooth muscle cell-specific marker proteins such as transgelin (SM22α), calponin, and alpha-smooth muscle actin (αSMA) [9,10,11,12]. Various signaling pathways, including Wnt and Notch, regulate VSMC gene expression by activating transcriptional factors or epigenetic modifications (Figure 1) [13,14].

**Figure 2:**
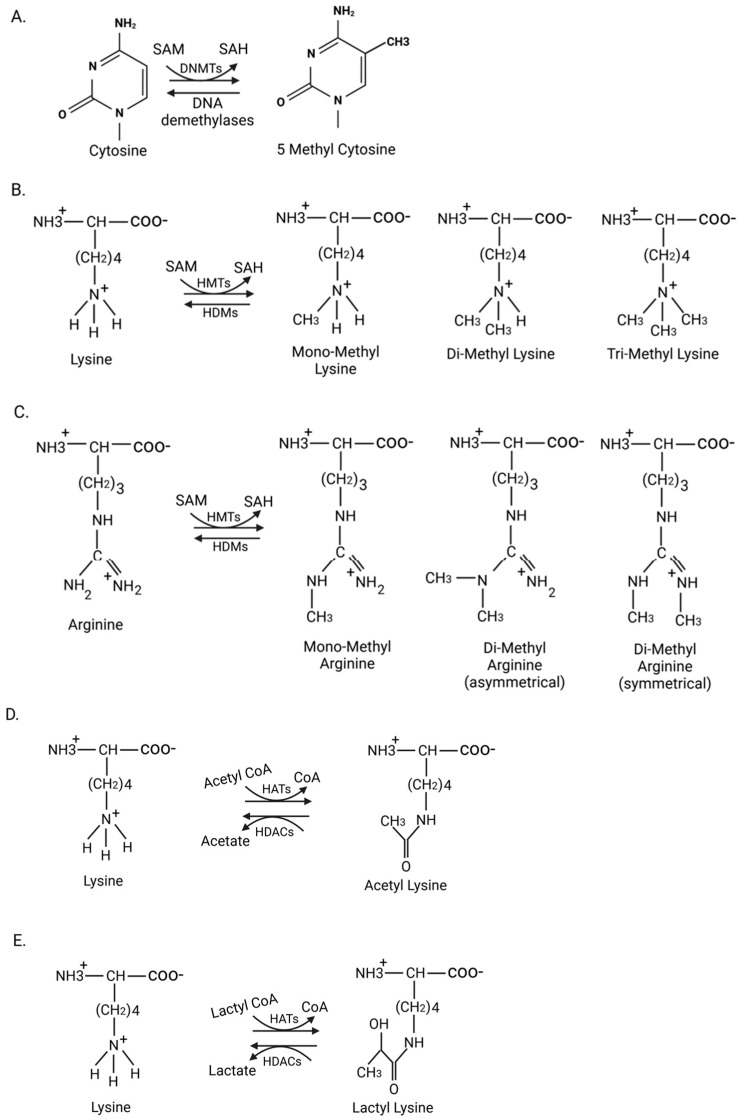
Various epigenetic modifications and their mediators. (**A**) DNA methylation: DNA methyltransferases (DNMTs) mediate methylation of cytosine residues on CpG islands, giving rise to 5-methylcytosine, which can be reversed by DNA demethylases. (**B**,**C**) Histone methylation: Histone methyl transferases (HMTs) add methyl groups to the epsilon amino group of lysine or the guanidino group of arginine on histones, resulting in either mono-, di-, or trimethyl states of lysine, and mono- and dimethyl states of arginine. These histone methyl groups are removed by histone demethylases (HDMs). (**D**,**E**) Histone acetylation and lactylation: Histone acetyltransferases (HATs) transfer acetyl and lactyl groups to the epsilon amino group of lysine on histones, resulting in histone acetylation and lactylation, respectively. These groups are removed by histone deacetylases (HDACs). SAM: S-adenosyl methionine; SAH: S-adenosyl homocysteine. Figure made in Bio-Render.

Epigenetic modifications generally include DNA methylation and histone modifications (methylation, acetylation, lactylation, etc.) (Figure 2), which regulate gene expression at the transcriptional level [15,16]. Additionally, non-coding RNAs act as epigenetic regulators that modulate gene expression both post-transcriptionally and at the transcriptional level [17]. Numerous studies have highlighted the role of epigenetics in the phenotypic switch of VSMCs to osteogenic cells. Epigenetic regulation has garnered significant attention as a therapeutic target in vascular calcification. This review explores the mechanisms mediating the epigenetic regulation of vascular calcification, ultimately leading to the osteogenic trans-differentiation of VSMCs.

## 2. DNA Methylation

DNA methylation involves the transfer of methyl groups to cytosine bases, resulting in the formation of 5-methylcytosine. This process is catalyzed by DNA methyltransferases (DNMTs) (Figure 2A). Based on their structure and function, DNMTs are classified as either maintenance enzymes, which include DNMT1, or de novo enzymes, which include DNMT3a and DNMT3b. DNMT1 copies the DNA methylation pattern from the parental strand to the newly synthesized strand during DNA replication, while DNMT3a and DNMT3b add methyl groups to unmethylated DNA [18,19].

DNA methylation has been extensively studied for its role in genome organization and the regulation of gene expression. A guanine residue follows cytosine residues that undergo methylation. These CpG dinucleotides are found in stretches of approximately 1000 base pair regions in the genome, known as CpG islands, which are often associated with gene promoter regions, and their methylation regulates gene expression, usually by inhibiting the binding of transcriptional factors [20,21]. Therefore, regulating the DNA methylation of CpG islands can influence the expression of genes that control processes such as cell differentiation, a crucial aspect of vascular calcification.

**Figure 3:**
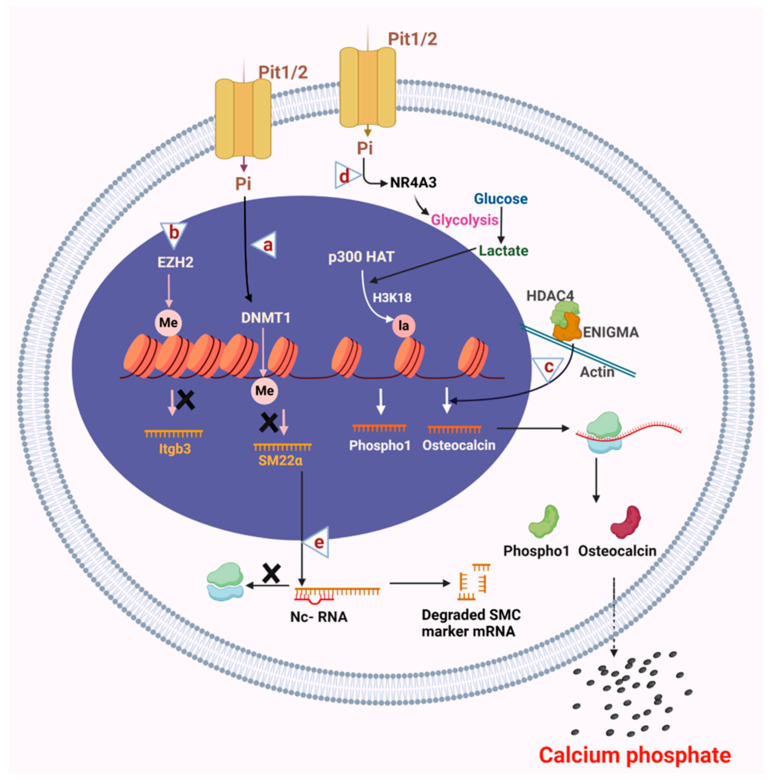
Epigenetic alterations in the VSMC osteogenic switch. (**a**) High phosphate conditions activate DNMT1, which causes methylation of CpG islands of DNA, leading to transcriptional repression of smooth muscle cell contractile protein SM22α. (**b**) Histone methyl transferase EZH2 binds to the promoter region of *Itgb3* and inhibits its expression, which promotes smooth muscle cell migration and calcification. (**c**) In the cytosol, histone deacetylase HDAC4 interacts with the cytosolic binding protein ENIGMA, an actin cytoskeleton binding protein. The HDAC4 and ENIGMA complex activates the expression of osteogenic protein osteocalcin through mechanosensing that results in calcification. (**d**) Increased expression of NR4A3 under high phosphate conditions causes an increase in glycolytic flux that leads to accumulation of lactate, which p300 HAT uses to mediate histone lactylation of the promoter regions of the *Phospho1* gene, thereby causing an increase in its expression and calcification. (**e**) Non-coding RNAs play an essential role in osteogenic differentiation by inhibiting the expression of VSMC contractile proteins by binding and degrading their mRNAs. Altogether, epigenetic mechanisms lead to the osteogenic differentiation of VSMCs that secrete calcium phosphate minerals into the extracellular space, leading to vascular calcification. ×: denotes inhibition of expression. Figure made in Bio-Render.

Vascular calcification is a clinical manifestation commonly linked with chronic kidney disease, which hinders renal filtration and accumulates various toxins and metabolites, including phosphate and indoxyl sulfate [22,23,24]. In vitro and ex vivo studies using human aortic VSMCs, and animal models have shown that high levels of phosphate and indoxyl sulfate induce calcification through alterations in DNA methylation [25,26]. High concentrations of phosphate also enhance the activity of DNMT1, leading to the upregulation of the osteogenic marker runt-related transcription factor 2 (*RUNX2/Cbfa1*) and the downregulation of the smooth muscle contractile protein SM22α, which results in a phenotypic switch of VSMCs and vascular calcification (Figure 3a). Conversely, inhibiting DNMT1 with procaine, a DNA demethylating agent that decreases 5-methylcytosine levels [27], prevents phosphate-induced calcification [25]. Similar results have been observed after treating cells and animal models with indoxyl sulfate. Indoxyl sulfate promotes vascular calcification by enhancing the expression of DNMT1, which leads to promoter methylation and decreases the expression of the anti-calcification protein Klotho. The pro-calcific effect of indoxyl sulfate is inhibited by treatment with DNMT1 inhibitor 5-aza-2′-deoxycytidine (5Aza-2dc) [28], that binds to DNA and inhibits its activity [26].

Aging is a significant risk factor for atherosclerosis [29,30]. Vascular aging is associated with remodeling of the extracellular matrix (ECM), primarily involving the breakdown of elastin, deposition of collagen, and cross-linking, which increases vascular stiffness [31,32]. Matrix stiffness has been shown to regulate the phenotype of vascular smooth muscle cells by influencing DNA methylation. VSMCs cultured on stiffer substrates that mimic increased ECM stiffness exhibit a reduced expression of DNMT1 compared to those on softer substrates. This decrease in DNMT1 leads to a diminished expression of smooth muscle cell marker proteins, such as alpha-smooth muscle actin, calponin, and SM22α, along with an increased expression of osteogenic marker proteins such as BMP2 and RUNX2, contributing to a phenotypic shift toward osteogenic cells, followed by calcification [33].

ECM stiffness regulates DNMT1 expression through mechanotransduction. This process involves sensing both external and internal mechanical cues (mechanosensing) and responding to these cues by inducing changes in gene expression and phenotypic transition [34,35]. For instance, increased ECM stiffness activates mechanosensory discoidin domain receptor 1 (DDR1) on VSMC cells, which triggers ERK-mediated phosphorylation of p53 and subsequent p53-mediated inhibition of DNMT1 expression, resulting in the transition of VSMCs to an osteogenic phenotype [36].

In summary, cardiovascular risk factors like chronic kidney disease and vascular aging change the functionally differentiated VSMC phenotype into a synthetic osteogenic phenotype by downregulating smooth muscle contractile proteins and upregulating synthetic proteins. These changes are driven by alterations in DNA methylation status in the promoter regions of the respective genes, leading to phenotypic changes and vascular calcification that ultimately contribute to atherosclerotic cardiovascular disease.

## 3. Histone Modifications

Histones are basic proteins that are rich in lysine and arginine. DNA, in association with histones, is organized into nucleosomes, which are the basic units of chromatin [37]. Besides DNA methylation, the post-translational modification of histones also regulates gene expression by controlling chromatin accessibility to transcriptional machinery. Histone-modifying enzymes, such as histone methyltransferases/demethylases and histone acetyltransferases/deacetylases, manage histone interactions with DNA by adding and removing methyl and acetyl groups. This process changes chromatin organization into either euchromatin, which is accessible to the binding of transcription factors and leads to gene expression, or heterochromatin, which is inaccessible to transcription factors and inhibits gene expression [38,39,40,41]. These changes in chromatin structure ultimately alter the VSMC phenotype. Among histone modifications, histone methylation, acetylation, and lactylation play crucial roles in the VSMC osteogenic phenotypic switch.

### 3.1. Histone Methylation

Histones undergo post-translational modifications on their lysine and arginine residues through histone methyltransferases (Figure 2B,C) [42,43]. These methyltransferases contain a catalytic SET domain that has been identified as a conserved region in three proteins of *Drosophila melanogaster*: Suppressor of variegation 3-9 (Su(var)3-9), Enhancer of zeste (E(z)), and Trithorax (Trx) [44]. Histone demethylases have a jumonji C (Jmjc) domain as their catalytic domain [45,46]. Three distinct states of histone methylation exist: mono-, di-, and tri-methylation. Depending on the position and state of methylation, histone modifications can either activate (e.g., H3K4, H3K36, H3K79) or repress (e.g., H3K9, H3K27, H4K20) transcription [47]. Numerous studies have identified histone methylation as a crucial regulator of the VSMC phenotypic switch in vascular calcification.

Inflammation is linked to vascular calcification. Pro-inflammatory cytokines released by macrophages are crucial in vascular calcification, as they promote the expression of osteogenic markers and induce phenotypic changes in VSMCs [48,49].

A recent study demonstrated that the proinflammatory cytokine interleukin-6 promotes vascular calcification partly by regulating histone methylation. Interleukin-6 (IL-6)/soluble interleukin-6 receptor (sIL-6R) encourages vascular calcification through an increase in phosphorylated STAT3 (signal transducer and activator of transcription 3), which, in combination with JMJD2B (histone demethylase), suppresses the repressive H3K9Me3 mark near the *RUNX2* promoter region, leading to an increased expression of RUNX2 and the transition of VSMCs to osteogenic cells [50].

The transition of VSMCs to an osteogenic phenotype is associated with increased expression of synthetic genes and decreased expression of contractile genes, followed by enhanced proliferation, migration, and calcification [51]. The histone methyltransferase Enhancer of zeste homolog 2 (EZH2) is a polycomb repressive complex 2 (PRC2) component that suppresses gene expression through repressive H3K27 dimethylation or trimethylation. Knockdown of EZH2 in mouse VSMCs resulted in reduced synthetic gene expression and decreased proliferation, migration, and calcification of VSMCs. It was also discovered that EZH2 interacts with the promoter of integrin *Itgb3* and represses its expression, leading to VSMC phenotypic transition towards a synthetic state (Figure 3b) [52]. Increased dimethylation of arginine 17 on histone H3 in low-density lipoprotein receptor-related protein 6 (LRP6) knockout mice is linked to dysregulated osteopontin expression, a glycoprotein produced by various cell types that plays a crucial role in vascular calcification by promoting the proliferation and migration of VSMCs [53].

### 3.2. Histone Acetylation

Histone acetyltransferases (HATs) and histone deacetylases (HDACs) maintain the homeostatic balance of histone acetylation. These enzymes catalyze the addition and removal of acetyl groups from histones, changing the chromatin structure and the expression of target genes (Figure 2D) [54,55]. Modifying the activity of HATs and HDACs is essential in regulating the VSMC phenotype.

Based on their homology to yeast HDACs, human HDACs are grouped into four classes: class I (HDAC 1,2,3,8), class II (HDAC 4,5,6,7,9,10), class III, which consists of sirtuins, and class IV, which includes HDAC 11. Based on the domain composition, class II is subdivided into class IIa (HDAC 4,5,7,9) and class IIb (HDAC 6,10) [56]. HDAC classes I and II and sirtuins have been shown to regulate vascular calcification. This review discusses the role of class I and II HDACs in vascular calcification. We refer to reviews by Pan et al. [57] and Wang et al. [58] for the role of sirtuins in vascular calcification.

The class I HDACs are predominantly localized in the nucleus and contain a deacetylase domain for histone deacetylation. Among class I HDACs, HDAC1 and HDAC2 inhibit vascular calcification, at least in part, through the regulation of autophagy. Autophagy is protective in vascular calcification by inhibiting VSMC phenotypic transition [59,60]. In rat VSMCs, HDAC1 inhibits vascular calcification by reducing the expression of histone demethylase LSD1 via regulation of H3K9 acetylation of the *LSD1* promoter region. Reduced LSD1 leads to increased autophagy and reduced vascular calcification [61]. HDAC2 overexpression attenuates the expression of osteopontin and osteocalcin. It inhibits the downregulation of αSMA and SM22α through increased autophagic flux in mouse models of CKD and human VSMCs treated with β-glycerophosphate [62].

Among class II HDACs, class IIa HDACs contain a deacetylase domain at their C-terminus and a MEF2 transcription factor binding domain at their N-terminus [63]. Class IIa HDACs shuttle between the nucleus and cytosol. Several important phosphorylation sites in class IIa HDACs regulate their nucleo-cytoplasmic localization. Once phosphorylated by specific kinases, the binding of 14-3-3 chaperones in the cytosol blocks their nuclear localization and allows cytoplasmic retention [64]. The cytosolic localization of HDAC4, mediated by a salt-inducible kinase (SIK), promotes vascular calcification by increasing osteocalcin expression [65]. Additionally, cytosolic HDAC4 engages with ENIGMA, a protein that binds to the actin cytoskeleton. The cytoskeleton connects to the nucleus via the linker of the nucleoskeleton and cytoskeleton complex (LINC), regulating gene expression through mechanosensing [66]. Speculation suggests the HDAC4-ENIGMA complex might influence VSMC gene expression via the LINC complex under osteogenic conditions (Figure 3c).

Our recent genome-wide meta-analysis study found that single nucleotide polymorphisms (SNPs) in the HDAC9 locus are associated with abdominal aortic calcification. Further, we showed that siRNA-mediated knockdown of HDAC9 in human VSMCs reduces RUNX2 expression and vascular calcification, while adenoviral expression of HDAC9 increased vascular calcification through increased expression of RUNX2 [67]. In addition, the loss of HDAC9 attenuated vascular calcification in the matrix Gla protein knockout (MGP KO) mouse model of vascular calcification, indicating that HDAC9 is a strong promoter of in vivo vascular calcification. Like HDAC4, HDAC9 shuttles between the nucleus and cytosol, and one study suggests that HDAC9 promotes vascular calcification by activating NF-κB [68]. However, the precise mechanisms by which HDAC9 regulates RUNX2 expression under osteogenic conditions remain incompletely elucidated.

### 3.3. Histone Lactylation

Besides its canonical role in adding acetyl groups, p300 acetyltransferase can also add lactyl groups in a process known as histone lactylation (Figure 2E). Lactate produced in glycolysis is a substrate to mediate histone lysine lactylation. This recently identified histone epigenetic modification plays a role in the metabolic regulation of gene expression [69].

Lactate is a byproduct of glycolysis, and VSMCs rely extensively on glycolysis, which results in lactate production [70]. Lactate promotes the VSMC synthetic phenotype with increased proliferation and migration [71]. Recent studies have emphasized the role of lactate in regulating VSMC calcification through histone lactylation. The transcription factor NR4A3 (nuclear receptor subfamily 4 group A member) regulates glucose metabolism. Under high phosphate stimulation, NR4A3 expression increases, promoting glycolysis and lactate production in VSMCs, which leads to enhanced p300 acetyltransferase-mediated lactylation of histone H3 (H3K18la) in the promoter region of *Phospho1*, a gene that facilitates VSMC calcification. This results in increased expression of Phospho1, which mediates the secretion of inorganic phosphate and is a potent inducer of vascular calcification (Figure 3d) [72]. Given that this is a relatively new field of study, more research is needed to understand the effects of histone lactylation globally on VSMC phenotype and function.

Overall, post-translational modification of histones, mediated by histone-modifying enzymes, plays a crucial role in regulating the VSMC phenotype. Cardiovascular risk factors such as inflammation and metabolic abnormalities promote vascular calcification through histone methylation and lactylation. On the one hand, some histone deacetylases, such as HDAC1 and HDAC2, inhibit vascular calcification by promoting autophagy, while others, such as HDAC4, promote it by regulating cytosolic non-histone proteins.

## 4. Non-Coding RNAs

Non-coding RNAs act as epigenetic regulators essential for post-transcriptional regulation (Figure 3e). They are classified into two main types based on size: long non-coding RNAs (lncRNAs) and small non-coding RNAs. Long non-coding RNAs are >200 nucleotides long. By contrast, small non-coding RNAs are <200 nucleotides long and include microRNAs (~20 nucleotides long), circular RNAs, small nuclear RNAs, and small nucleolar RNAs. Among small non-coding RNAs, microRNAs have been extensively studied for their role in vascular calcification [73,74,75]. The present review addresses the role of long non-coding RNAs and microRNAs in promoting vascular calcification.

### 4.1. Long Non-Coding RNAs

Long non-coding RNAs (lncRNAs) are defined as non-protein coding RNAs longer than 200 nucleotides. Their function depends on their binding target: lncRNAs can modulate the stability of DNA/RNA or can bind and change chromatin complex structures in the form of epigenetic regulation [76]. lncRNAs modulate the effects of many calcification factors, including the osteogenic effects of high-phosphate conditions [77,78,79], the inhibitory effect of antioxidants [80], and the calcifying effects of mineralocorticoid receptors [81]. The mechanisms by which lncRNAs modulate calcification are similarly diverse with wide-ranging effects on inflammation, autophagy, and cellular senescence, amongst other processes. Most pro-calcifying lncRNAs are found to act through miRNA sponging, in which the lncRNA binds a target miRNA to inactivate it. The lncRNA H19, which is imprinted maternally, sponges several microRNAs to increase inflammatory TLR3 signaling and Erk1/2 phosphorylation, and ultimately cause Capitalize RUNX2 upregulation and calcification [82,83,84]. Similarly, the lncRNA Metastasis Associated Lung Adenocarcinoma Transcript 1 (MALAT1) promotes RUNX2-mediated calcification by sponging miR-30c, while LINC00458 (aka ES3) promotes calcification and senescence through direct sponging of miR-34c-5p and resulting increases in Bcl-2 modifying factor [85,86].

Other lncRNAs promote vascular calcification through their effects on cellular senescence. The pro-calcification lncRNAs small nucleolar RNA host gene 1 (SNHG1), ES3, and BMF antisense 1 (BMF-AS1) regulate microRNAs, transcription factors, and other proteins to promote dysregulated autophagy, thereby leading to senescence and calcification [86,87,88].

In addition to influencing master regulators of vascular calcification, lncRNAs can reduce calcification through various signaling pathways. Inhibition of vascular calcification via lncRNAs such as small nucleolar RNA host gene 29 (SNHG29) and long intergenic RNA-erythroid pro-survival (lincRNA-EPS) has been associated with increased Klotho signaling and decreased Smad3, Wnt, and β-catenin signaling [89,90]. lncRNA GAS5 may inhibit vascular calcification by promoting PTEN (phosphatase and tensin homolog) signaling and suppressing VSMC phenotypic switching, proliferation, and migration [91,92].

As assays evolve and annotations become increasingly available for genes and transcripts, searches for influential lncRNAs have increased in scope and produced large sets of lncRNAs associated with vascular calcification. Multiple studies have conducted transcriptome-wide searches for gene transcripts influenced by calcifying conditions [77,93,94]. These studies have generated regulatory networks to identify lncRNAs that influence important proteins in calcification pathways, such as the transcription factors FOXO1 and SNAI2 [95]. Thus, these assays provide additional lncRNAs that identify new molecular mechanisms of calcification and potential therapeutic targets for modifying signaling pathways, transcription factors, and other mechanisms important to vascular calcification.

In summary, lncRNAs are a class of epigenetic regulators known to target many well-known signaling pathways and cellular phenomena in the vasculature. Though it is a newer area of investigation, targeting lncRNAs may represent a powerful class of epigenetic therapies in vascular calcification.

### 4.2. Small Non-Coding RNAs

MicroRNAs have been extensively studied among small non-coding RNAs for their role in vascular calcification [96]. MicroRNAs are approximately 22 nts long and mostly bind to the 3′UTR of target mRNA, leading to their degradation and inhibition of gene expression. Several miRNAs regulate the VSMC osteogenic transition; some promote, while others inhibit. Some well-studied miRNAs promoting vascular calcification include miR-32, miR-34a, and miR-155. Among these, miR-32 has been shown to promote vascular calcification through various mechanisms, including inhibition of PTEN, which activates PI3K/AKT signaling, resulting in increased expression and phosphorylation of osteogenic mediator RUNX2 [97]. A recent study demonstrated that treating high-phosphate-induced VSMCs with the Bushen Huoxue Formula, a traditional Chinese medicine, reduced exosomal miR-32, enhanced PTEN expression, and reduced osteogenic mediator proteins BMP-2 and RUNX2 [98]. Additionally, miR-32 promotes vascular calcification by inhibiting autophagy [99] and by increasing the expression of proinflammatory mediator TNFα [100]. Vascular miR-34a expression increases with aging and is essential in age-mediated vascular senescence. Increased miR-34a levels were associated with increased secretion of pro-inflammatory cytokine IL-6 and increased arterial inflammation and vascular calcification [101]. Further, miR-34a reduces the expression of vascular calcification inhibitors Axl (AXL Receptor Tyrosine Kinase) and SIRT1 (Sirtuin 1) [102]. Indoxyl sulfate has been shown to induce miR-155, downregulating the anti-calcific protein matrix Gla protein (MGP), thereby increasing vascular calcification [103]. Further, depletion of miR-155 inhibits vascular calcification by inhibiting AKT phosphorylation and FOXO3a degradation [104]. Several other miRNAs exert an inhibitory effect on vascular calcification. For example, endothelial-derived miR-204-5p inhibits vascular calcification by reducing the expression of osteogenic mediators RUNX2 and BMP2 [105]. Another miRNA, mir-133a, inhibits osteogenic differentiation and VSMC calcification by downregulating the expression of RUNX2 [106].

Data supporting the vital role of microRNAs in vascular calcification continue to emerge. Each miRNA likely has more than one target, making the biology of miRNAs in vascular calcification multi-factorial. Moreover, the fact that miRNAs are expressed in a tissue-specific manner but are also secreted (i.e., in exosomes) confers the ability for miRNAs to exert effects in an autocrine, paracrine, and long-range endocrine fashion that adds to the complex downstream effects of miRNAs in vivo.

## 5. Targeting Epigenetic Regulators to Treat Vascular Calcification

Thus far, no therapies have been approved to treat vascular calcification. Preventative strategies include lifestyle modifications to prevent risk factors such as diabetes, hypertension, and chronic kidney disease. When prevention fails, interventionalists can offer methods to treat stenotic lesions of vascular calcification. For instance, rotational atherectomy has been used to treat calcified coronary and peripheral lesions through the high-speed rotation of a diamond-tipped burr that fractures and debulks calcium plaque. Intravascular lithotripsy is another technique that disrupts calcium plaques using ultrasonic shock waves [107,108]. While these methods help treat patients with established vascular calcium deposits, preventative treatments for calcification are urgently needed to prevent adverse cardiovascular outcomes.

Several intervention studies have investigated the potential efficacy of drugs to attenuate vascular calcification. A recent review summarized the results of several randomized clinical trials on calcification attenuation [109]. Aged garlic extract (AGE) has been shown to inhibit lipid accumulation in the thoracic aorta and prevent the phenotypic switch of VSMCs [110]. In multiple studies, AGE treatment significantly attenuated coronary artery calcification progression and subclinical atherosclerosis [111,112,113,114,115,116]. Statins, which are hydroxy methyl glutaryl-CoA (HMG-CoA) reductase inhibitors that inhibit cholesterol biosynthesis in the liver, do not attenuate calcification [117,118,119,120,121,122,123,124,125,126,127,128,129]. Vitamin K is a co-factor for the gamma-glutamyl carboxylase enzyme that catalyzes the carboxylation of glutamate residues in matrix Gla protein [130], whose carboxylation status is critical for its anti-calcific properties [131]. Vitamin K treatment may reduce the progression of coronary artery calcification [132,133], but larger-scale clinical trials are needed to define these potential benefits. Lifestyle changes, including strict dietary control and physical activity, did not attenuate coronary calcification [134,135,136,137]. In a trial using nifedipine, an anti-hypertensive agent, the authors found a significant attenuation in coronary calcium [138]; however, another study with the same drug showed no effect [139]. Omega-3 fatty acids [140,141], hypoglycemic agents to reduce blood sugar levels [142,143], hormone replacement therapies [144,145,146], and treatment with inhibitors of platelet aggregation, such as sarpogrelate [147] and cilostazol [148], have all been studied to assess the impact on coronary artery calcification and non-calcified atherosclerotic plaque with these trials having mixed results and for the most part not showing a significant reduction in calcified plaque.

Since transforming VSMCs to osteogenic cells is a crucial step in calcification, therapies that prevent this phenotype switch may help treat vascular calcification. As discussed above, in vitro and in vivo evidence from many studies suggests that epigenetic regulators of VSMC osteogenic phenotype switch may be an excellent therapeutic strategy for preventing (or slowing the progression of) vascular calcification. For example, inhibition of DNMTs attenuates osteogenic differentiation under high phosphate conditions that model chronic kidney disease. The FDA has approved the DNMT inhibitor 5-azacytidine to treat myelodysplastic syndromes, and it could be a potential therapeutic agent for inhibiting osteogenic differentiation [149]. In addition to DNMTs, HMTs promote osteogenic differentiation of VSMCs. Tazemetostat is another drug approved by the FDA for treating epithelioid sarcoma and is an inhibitor of histone methyltransferase EZH2 [150]. Histone acetylation is vital in VSMC osteogenic differentiation, and class IIa histone deacetylases promote osteogenic differentiation. Four HDAC inhibitors, vorinostat [151,152], romidepsin [153], panobinostat [154], and belinostat [155], have been approved by the FDA for treating hematologic cancers. Further study is needed to determine if these FDA-approved therapies can be used as an alternative indication of vascular calcification. However, the HDAC inhibitors are non-selective and inhibit more than one class of HDACs. Since class I HDACs protect against vascular calcification while class II HDACs promote it, non-specific HDAC inhibitors may not be ideal for treating it. Therefore, there is a need to develop more selective pharmacological agents to inhibit HDACs in calcification.

Many in vitro studies have shown that vascular calcification can be mitigated by correcting perturbations in lncRNA levels. The downregulation of H19 reduces calcification in vitro through either MAPK or NF-κB signaling reduction [83,84]. Similarly, inhibition of lncRNAs TUG1 [156] and MALAT1 [85] reduced calcification through suppression of RUNX2 expression and FAS-AS1 inhibition reduced calcification by suppressing inflammatory response to hyperphosphatemia [157]. Restoration of the anti-calcifying lncRNAs GAS5 [79] and ANCR [78] has been shown to mitigate vascular calcification by increasing signaling through the PTEN pathway or enhancing autophagy. ANCR is particularly interesting and has been tested as an agent for reducing calcification in vascular graft tissues. By supplying exosomes containing ANCR targeted at Gli+ (smooth muscle progenitor) cells, osteogenic differentiation was mitigated, and the development of calcification was suppressed [158]. Similarly, small non-coding RNAs, such as microRNAs, affect vascular calcification by inhibiting PTEN, autophagy, or attaining a senescence-associated secretory phenotype of smooth muscle cells that secrete proinflammatory cytokines that aid in VSMC calcification [97,99,101]. Alternatively, some miRNAs inhibit calcification by reducing the expression of osteogenic markers such as RUNX2 and BMPs [105,106]. Future therapies for non-coding RNAs may be promising, as precisely designed antisense oligonucleotides can specifically target non-coding RNAs. However, various issues must be addressed to put these therapies into practice, such as oligonucleotide stability, targeted delivery to tissue (vasculature) and the possibility of off-target effects [159].

In conclusion, many primary risk factors for cardiovascular diseases—including chronic kidney disease, inflammation, vascular stiffness, and metabolic abnormalities—contribute to the osteogenic phenotypic switch of vascular smooth muscle cells by altering the epigenetic landscape, resulting in vascular calcification. With no currently approved therapies, attenuating vascular calcification remains a challenge and an opportunity for future research endeavors.

## Abbreviations

The following abbreviations are used in this manuscript:

AGE	Aged garlic extract
αSMA	Alpha-smooth muscle actin
ALP	Alkaline phosphatase
ANCR	Angelman syndrome chromosome region
5aza-2dc	5-aza-2′-deoxycytidine
BMPs	Bone morphogenic proteins
CVDs	Cardiovascular diseases
DDR1	Discoidin domain receptor 1
DNMTs	DNA methyltransferases
ECM	Extracellular matrix
ERK	Extracellular signal-regulated kinase
EZH2	Enhancer of zeste homolog 2
FOXO	Forkhead box O
GAS5	Growth arrest-specific 5
HATs	Histone acetyltransferases
HDACs	Histone deacetylases
HMTs	Histone methyl transferases
IL-6	Interleukin-6
LINC	Linker of the nucleoskeleton and cytoskeleton complex
lncRNAs	Long non-coding RNAs
lincRNA-EPS	Long intergenic RNA-erythroid pro-survival
LRP6	Lipoprotein receptor-related protein 6
MALAT1	Metastasis associated lung adenocarcinoma transcript 1
MAPK	Mitogen-activated protein kinase
NF-κB	Nuclear factor kappa B
NR4A3	Nuclear receptor subfamily 4 group A member
PRC2	Polycomb repressive complex 2
PTEN	Phosphate and tensin homolog
RUNX2	Runt-related transcription factor 2
sIL-6R	Soluble interleukin-6 receptor
SM22α	Smooth muscle protein 22-alpha
SNAI2	Snail family transcriptional repressor 2
SNHG1	Small nucleolar RNA host gene 1
STAT3	Signal transducer and activator of transcription 3
TLR3	Toll like receptor 3
TUG1	Taurine upregulated 1
VSMCs	Vascular smooth muscle cells
Wnt	Wingless integrated
